# LigD: A Structural Guide to the Multi-Tool of Bacterial Non-Homologous End Joining

**DOI:** 10.3389/fmolb.2021.787709

**Published:** 2021-11-25

**Authors:** Benhur Amare, Anthea Mo, Noorisah Khan, Dana J. Sowa, Monica M. Warner, Andriana Tetenych, Sara N. Andres

**Affiliations:** ^1^ Department of Biochemistry and Biomedical Sciences, McMaster University, Hamilton, ON, Canada; ^2^ Michael G. DeGroote Institute for Infectious Disease Research, McMaster University, Hamilton, ON, Canada

**Keywords:** LigD, non-homologous end joining, DNA double-strand break, protein structure and function, ligase, polymerase, phosphoesterase

## Abstract

DNA double-strand breaks are the most lethal form of damage for living organisms. The non-homologous end joining (NHEJ) pathway can repair these breaks without the use of a DNA template, making it a critical repair mechanism when DNA is not replicating, but also a threat to genome integrity. NHEJ requires proteins to anchor the DNA double-strand break, recruit additional repair proteins, and then depending on the damage at the DNA ends, fill in nucleotide gaps or add or remove phosphate groups before final ligation. In eukaryotes, NHEJ uses a multitude of proteins to carry out processing and ligation of the DNA double-strand break. Bacterial NHEJ, though, accomplishes repair primarily with only two proteins–Ku and LigD. While Ku binds the initial break and recruits LigD, it is LigD that is the primary DNA end processing machinery. Up to three enzymatic domains reside within LigD, dependent on the bacterial species. These domains are a polymerase domain, to fill in nucleotide gaps with a preference for ribonucleotide addition; a phosphoesterase domain, to generate a 3′-hydroxyl DNA end; and the ligase domain, to seal the phosphodiester backbone. To date, there are no experimental structures of wild-type LigD, but there are x-ray and nuclear magnetic resonance structures of the individual enzymatic domains from different bacteria and archaea, along with structural predictions of wild-type LigD via AlphaFold. In this review, we will examine the structures of the independent domains of LigD from different bacterial species and the contributions these structures have made to understanding the NHEJ repair mechanism. We will then examine how the experimental structures of the individual LigD enzymatic domains combine with structural predictions of LigD from different bacterial species and postulate how LigD coordinates multiple enzymatic activities to carry out DNA double-strand break repair in bacteria.

## Introduction

To repair a lethal DNA double-strand break (DSB), living organisms use two central pathways: homologous recombination (HR) and non-homologous end joining (NHEJ). HR is the ubiquitous DNA repair pathway that provides high-fidelity repair of DNA DSBs. HR is active during cell division in eukaryotes, primarily taking place during the S and G2 phases of the cell cycle, as it requires a template strand of DNA in order to complete its repair. This template strand is normally DNA from a sister chromatid, which is more readily available in the late stages of cell division before mitosis [reviewed in: Li and Heyer, 2008; Brandsma and Gent, 2012; Wright et al., 2018)]. Prokaryotic organisms, similar to eukaryotes, use HR during periods of active replication, where the replicated DNA can serve as a template for repair (reviewed in: Kowalczykowski et al., 1994; Ayora et al., 2011). However, bacterial metabolism slows during sporulation, latent infections, desiccation, and the stationary phase of growth, where a sister chromosome would not be present, suggesting an alternative DSB repair pathway is required, such as NHEJ (Pitcher et al., 2007c; Moeller et al., 2007; Stephanou et al., 2007; Brzostek et al., 2014). The first hints of an NHEJ repair pathway in bacteria came from *in silico* studies that identified homologs of the eukaryotic NHEJ repair proteins Ku70/80 and ATP-dependent DNA ligases in some bacteria (Aravind and Koonin, 2001; Weller et al., 2002). Further studies have shown that NHEJ is not ubiquitous in bacteria, but is found in approximately 20–25% of the kingdom, with a slight trend to species containing larger genomes with higher GC content and slower growth rates (McGovern et al., 2016). Many *in vivo* and *in vitro* studies have used the model organisms *Pseudomonas aeruginosa, Mycobacterium tuberculosis*, *Mycobacterium smegmatis*, and *Bacillus subtilis* to further establish the presence of the NHEJ repair pathway in a subset of bacteria (Weller et al., 2002; Della et al., 2004; Gong et al., 2004, 2005; Zhu and Shuman, 2005a, 2005b; Korycka-Machala et al., 2006; Pitcher et al., 2007c; Moeller et al., 2007; Stephanou et al., 2007; Zhu and Shuman, 2007). *B. subtilis* and *M. smegmatis* cells in stationary phase, carrying gene deletions of the NHEJ machinery, had reduced cell survival and exhibited sensitivity to ionizing radiation, illuminating a role for NHEJ *in vivo* (Stephanou et al., 2007; de Vega, 2013). The same gene deletions of the NHEJ machinery in *M. smegmatis* also resulted in cells highly sensitive to desiccation, a state that induces DNA DSBs in bacteria (Pitcher et al., 2007c). These results suggested that NHEJ plays a role for bacteria in quiescent states (Leggett et al., 2012; Palomino and Martin, 2014).

**FIGURE 1:**
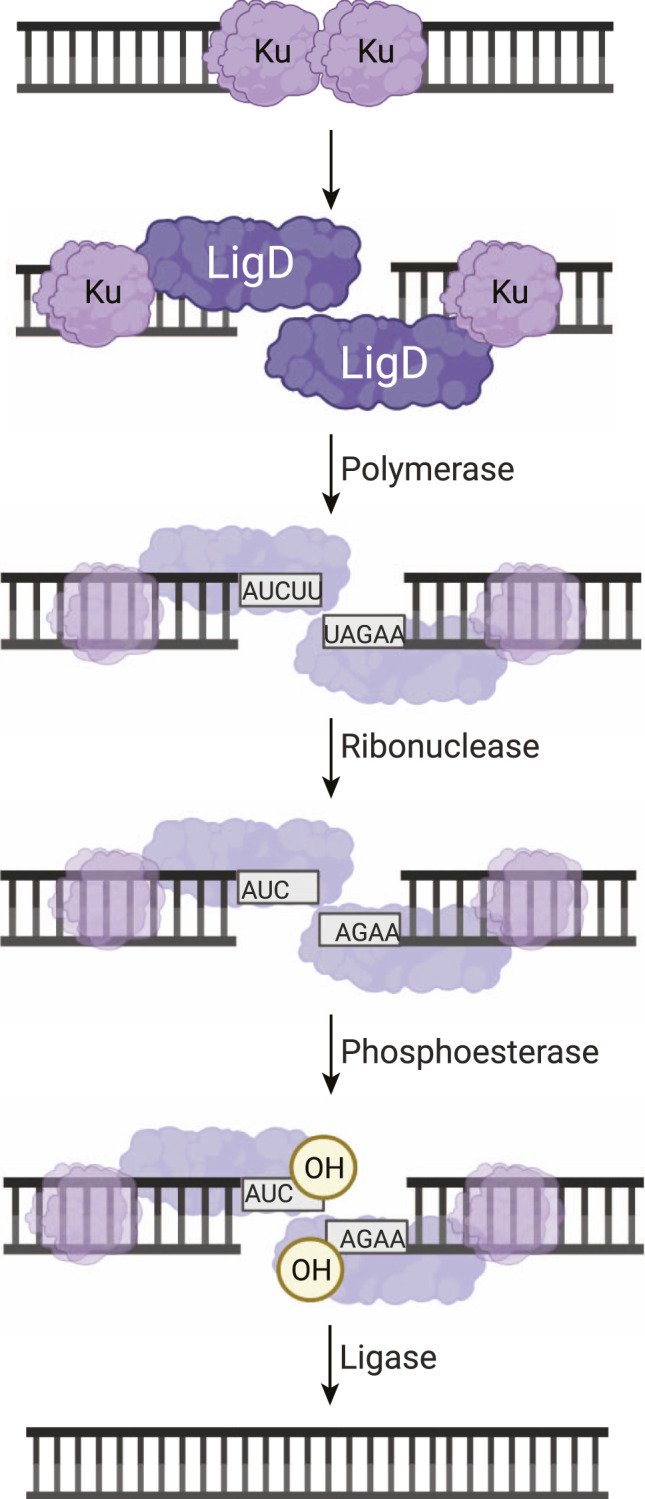
Non-homologous end joining repair of a DNA double-strand break in bacteria. Ku homodimers bind the DSB, recruiting LigD to the DNA ends. The DNA ends can be processed by LigD to add nucleotides through the LigD polymerase domain. LigD can also remove ribonucleotides or convert a 3′-phosphate to a 3′-hydroxyl via the LigD phosphoesterase domain. Once the DNA ends consist of a 5′-phosphate and a 3′-hydroxyl, the LigD ligase domain seals the phosphodiester backbone, repairing the DSB. Image created with Biorender.com.

NHEJ requires binding of the DNA DSB followed by processing of the DNA ends to yield a 5′-phosphate and 3′-hydroxyl, leading to a chemically competent state for final ligation of the phosphodiester backbone. To achieve DNA DSB repair, prokaryotic NHEJ is dependent on the Ku70/80 homolog, Ku, and a multi-functional ATP-dependent ligase, LigD (Figure 1) (Weller et al., 2002; Bowater et al., 2006). Ku recognizes, binds to, and bridges across the ends of the double-strand break, protecting the DNA ends from further damage (Shuman and Glickman, 2007; Öz et al., 2021). Ku homologs from *B. subtilis* and *P.aeruginosa* have reported lyase activity, useful for processing of the DSB ends, by removing abasic sites that may interfere with repair (De Ory et al., 2014). The critical function of Ku, though, is the recruitment of LigD to the DNA break. While eukaryotic NHEJ uses a plethora of polymerases, nucleases, kinases and more to process the DNA ends (Zhao et al., 2020), prokaryotic NHEJ has LigD. LigD is the multi-tool of prokaryotic NHEJ, carrying out polymerase, phosphoesterase, nuclease, and ligase activities (Della et al., 2004; Zhu and Shuman, 2005a; Zhu and Shuman, 2005b; Zhu and Shuman, 2007; Gong et al., 2005; Shuman and Glickman, 2007). Each of these enzymatic functions must be regulated and coordinated to achieve repair, depending on the type of damage found at the double-strand break. To better understand the mechanism of how LigD directs these repair activities, we will examine the current literature on LigD from a structural perspective. We will consider experimental atomic structures of the individual enzymatic domains and their insights on LigD mechanism and combine these findings with the *in silico* atomic models of LigD (Jumper et al., 2021; Mirdita et al., 2021) from *M. tuberculosis, P. aeruginosa,* and *B. subtilis*.

### Ku, The LigD Activator

**FIGURE 2:**
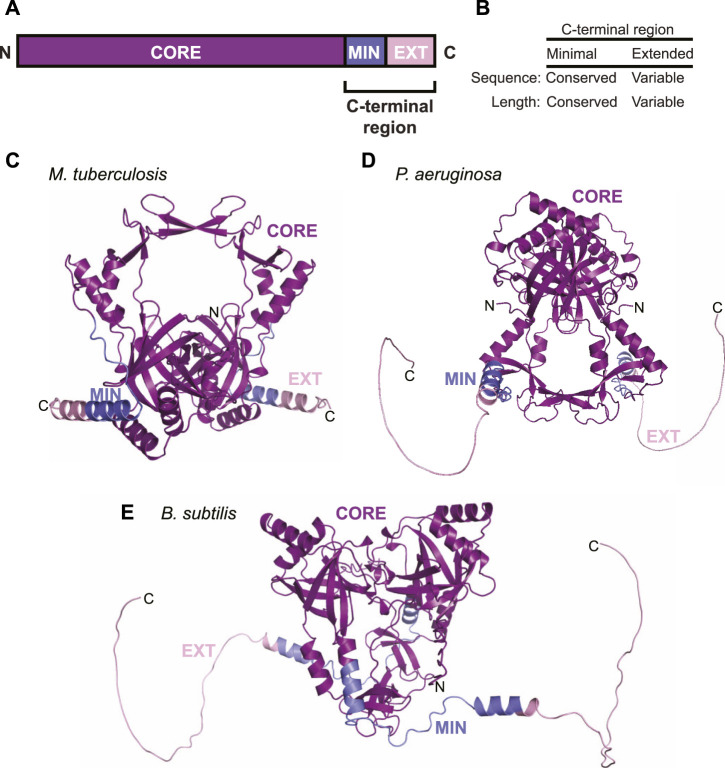
Domain arrangement and *in silico* models of Ku. **(A)** Domain arrangement of Ku in bacteria capable of non-homologous end joining. MIN, minimal C-terminus; EXT, extended C-terminus. **(B)** Comparison of conservation of sequence and length between the minimal and extended Ku C-terminus. **(C–E)** *In silico* models of **(C)** *M. tuberculosis*, **(D)** *P. aeruginosa* and **(E)** *B. subtilis* Ku homodimers, predicted by ColabFold (Mirdita et al., 2021). Purple, core domain; blue, minimal C-terminus; pink, extended C-terminus.

In order for LigD to participate in NHEJ, it requires its binding partner, Ku. The prokaryotic Ku protein is a homodimer of approximately 30–40 kDa, consisting of a core domain conserved with the eukaryotic Ku homolog, and a C-terminus unique to bacteria (McGovern et al., 2016). The C-terminus of Ku can be further subdivided into a minimal and an extended region. The minimal C-terminal region is conserved amongst bacteria, while the extended C-terminal region has low sequence conservation and is highly variable in length between organisms (Figures 2A,B) (Kushwaha and Grove, 2013b; McGovern et al., 2016). The core domain is predicted to form a ring-shaped structure that encircles DNA, much like eukaryotic Ku70/80, based on high sequence homology, and various *in silico* atomic structures from *B. subtilis* and *M. tuberculosis* (McGovern et al., 2016; Jumper et al., 2021; Öz et al., 2021). The structure of the C-terminus, though, is likely variable. Disorder predictions indicate the C-terminus is an intrinsically disordered region (Oates et al., 2013), which correlates with findings from small-angle x-ray scattering studies of *B. subtilis* Ku (McGovern et al., 2016). We carried out *in silico* modelling of Ku homodimers using the ColabFold notebook, which uses the AlphaFold algorithm (Jumper et al., 2021; Mirdita et al., 2021). The resulting predictions of Ku shows that the shorter C-terminus of *M. tuberculosis* Ku may take on some structure, with alpha helices binding the C-terminus to the core, interspersed with disordered loops (Figure 2C). *P. aeruginosa* and *B. subtilis* Ku, though, have longer, disordered C-termini, corroborated by ColabFold’s low confidence score in positioning the modelled C-termini (Figures 2D,E) (Jumper et al., 2021; Mirdita et al., 2021; Öz et al., 2021).

Ku recruits LigD to the DSB and stimulates both ligase and polymerase activities of LigD (Aravind and Koonin, 2001; Weller et al., 2002; Della et al., 2004; Zhu and Shuman, 2010; McGovern et al., 2016; Öz et al., 2021). The Ku C-terminus of *B. subtilis* and *P. aeruginosa* Ku are credited with stimulating ligation, while the Ku *B. subtilis* minimal C-terminal region is needed to interact with LigD (Zhu and Shuman, 2010; McGovern et al., 2016; Öz et al., 2021). The extended C-terminal region of *B.subtilis* and *M. smegmatis* Ku can bind supercoiled DNA (Kushwaha and Grove, 2013a; McGovern et al., 2016), while the *B. subtilis* Ku extended C-terminal region also restricts movement along dsDNA (McGovern et al., 2016; Öz et al., 2021). It is likely this combination of interacting with LigD and binding to DNA that permits Ku to tether LigD and then stimulate LigD polymerase and ligase activities at the DSB.

### An Overview of LigD Functional Domains

**FIGURE 3:**
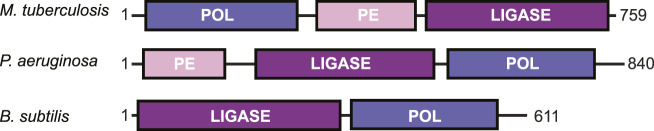
Domain arrangement of LigD in bacteria capable of non-homologous end joining. POL, polymerase domain; PE, phosphoesterase domain; LIGASE, ligase domain.

DNA DSBs are rarely a clean break with complementary ends containing a 5′-phosphate and 3′-hydroxyl group. Depending on the DNA damage, there may be gaps in the nucleotide sequence or a phosphate group in place of a hydroxyl at the 3′-end of the DNA (Andres et al., 2015). To repair the DNA to ligation-competent 5′-phosphate and 3′-hydroxyl ends, LigD possesses multiple enzymatic functions. These activities are contained in conserved structural domains that are briefly: the polymerase domain (POL) for the addition of nucleotides; the phosphoesterase/nuclease domain (PE) to convert 3′-phosphate groups to hydroxyl groups; and the ligase domain (LIG) to seal the phosphodiester backbone of the DNA (Weller and Doherty, 2001; Weller et al., 2002; Gong et al., 2005; Pitcher et al., 2005; Zhu and Shuman, 2005a, Zhu and Shuman, 2005b; Zhu and Shuman, 2010; de Vega, 2013). These domains are conserved across bacterial species, however not every domain is always found within a LigD homolog, nor maintained in the same primary structure (Figure 3). For example, *B. subtilis* LigD lacks the PE domain, with only the LIG domain at the N-terminus and the POL domain at the C-terminus (de Vega, 2013). Meanwhile, *M. tuberculosis* and *P. aeruginosa* possess all three domains, but rotate the sequential order of each domain between organisms (Pitcher et al., 2005; Zhu and Shuman, 2005a; Zhu and Shuman, 2005b; Zhu and Shuman, 2006). Given the changes in the sequential order, it remains to be seen how LigD coordinates the processing and ligation of complex DNA end damage in the context of the wild-type protein. Ku may play a role in coordination, as *M. tuberculosis* Ku directly interacts with the POL domain and weakly interacts with the LIG domain (Della et al., 2004; Pitcher et al., 2005; Wright et al., 2010). However, atomic structures resolved by x-ray crystallography and nuclear magnetic resonance of the POL, PE, and LIG domains in different enzymatic states, combined with *in silico* predictions of LigD can provide insights into the unique and complementary functions of each domain within LigD. A list of the experimentally determined structures of LigD discussed in this review can be found in Supplementary Table S1.

### The Ligase Domain

The LigD LIG domain maintains the conserved ATP-dependent ligation mechanism found in all DNA ligases, which uses three nucleotidyl transfer reactions to seal a DSB (Weller et al., 2002). First, a nucleophilic attack by the catalytic lysine on the α-phosphorus of ATP creates a covalent ligase-AMP intermediate and releases pyrophosphate as a by-product. The resulting configuration leaves the 5′-phosphate at the DNA break site primed to attack the α-phosphorus of the ligase-AMP intermediate. This reaction transfers the AMP moiety onto the 5′ end of the DNA, creating a DNA-adenylylate intermediate. In the final step, the phosphodiester bond is formed after the 3′-hydroxyl attacks the DNA-adenylylate, releasing the AMP and sealing the DNA backbone (Tomkinson et al., 2006). Interestingly, while the ligase mechanism is typically associated with sealing a nick between the ends of deoxyribonucleotides, the LIG domain in *P. aeruginosa, M. tuberculosis* and *Agrobacterium tumefaciens* preferentially seals nicks with a 3′-monoribonucleotide (Zhu and Shuman, 2008; Unciuleac et al., 2019).

**FIGURE 4:**
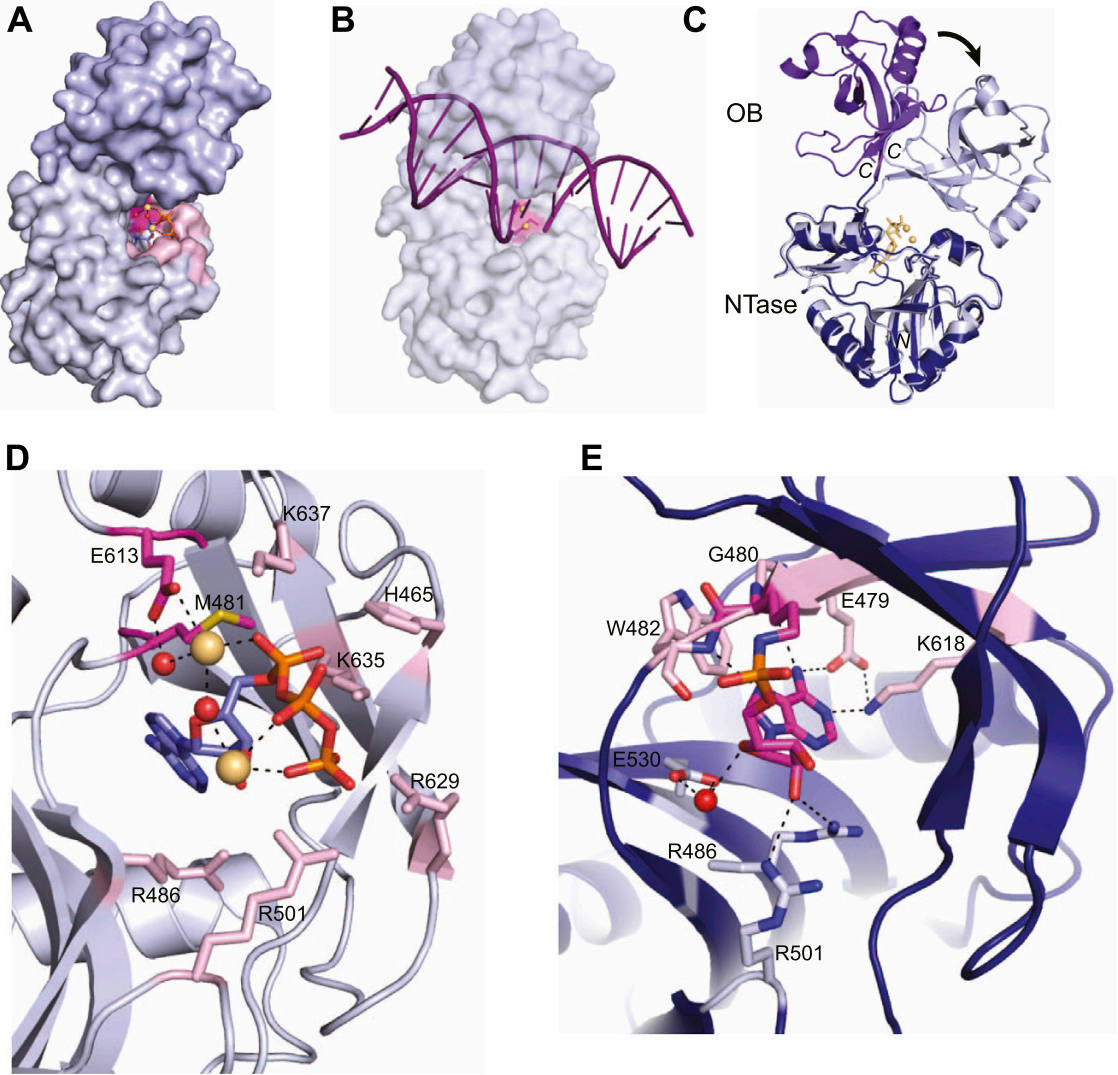
Atomic structures of the LigD ligase domain in open and closed conformations from *M. tuberculosis*. **(A)** Surface representation of LigD ligase domain (PDB 6NHZ) in the closed conformation. ATP (blue and orange sticks), magnesium (yellow spheres) and water molecules (red) are in the active site, surrounded by amino acids hydrogen bonding to the ATP. The active site (highlighted pink) with the catalytic lysine in magenta, is located within the NTase domain (grey) and capped by the OB domain (purple). **(B)** Surface representation of LigD ligase domain as in **(A)**, with a nicked DNA substrate (purple). The NTase domain of human LigIV, bound to a DNA nick (PDB 6BKG) was aligned with the NTase domain of LigD (RMSD = 2.7 Å) to illustrate the possible location of the DNA nick near the active site (pink). **(C)** Structural alignment of the open conformation (PDB 1VS0, NTase dark blue, and OB purple) and closed conformation (PDB 6NHZ, NTase grey, and OB light purple) of the LigD ligase domain. Black arrow indicates rotation. Yellow sticks and spheres are ATP and magnesium from the closed conformation. **(D)** Active site of the LigD ligase closed conformation. The catalytic Lys481 has been mutated to methionine (magenta backbone). Amino acids interacting with the catalytic magnesium have a magenta backbone; amino acids that stabilize the active site through hydrogen bonding have a pink backbone. ATP, purple and orange sticks; magnesium, yellow spheres; water molecules, red spheres; black dashed lines indicate hydrogen bonding. **(E)** Active site of the LigD ligase domain in open conformation. The LIG-AMP intermediate has a magenta backbone, while amino acids interacting with the ribose sugar have a grey backbone and amino acids interacting with the adenine base have a pink backbone. Black dashed lines indicate hydrogen bonding Red sphere, water molecule. Figures generated with The PyMOL Molecular Graphics System, Version 2.0 Schrödinger, LLC.

Current x-ray crystal structures of the LIG domain in the Protein Data Bank are from *M. tuberculosis* LigD and capture both the pre-adenylylation state (PDB 6NHZ) (Unciuleac et al., 2019) and the covalent LIG-AMP intermediate (PDB 1VS0) (Akey et al., 2006), providing mechanistic insight on the initiation of ligation. The LIG domain is sub-divided into an N-terminal nucleotidyltransferase (NTase) domain and an oligonucleotide binding (OB) domain, which is most similar to human LigI (Figure 4A) (Akey et al., 2006; Unciuleac et al., 2019). The NTase domain forms a nucleotide binding pocket and houses the catalytic lysine that forms the ligase-AMP intermediate (Figures 4A,C) (Akey et al., 2006; Unciuleac et al., 2019). A linker connects the NTase domain to the OB domain, which binds the nicked DNA and positions the DNA into the active site for repair (Akey et al., 2006; Unciuleac et al., 2019)

In the pre-adenylylation state, the OB domain forms a cap over the ATP-binding pocket in the NTase domain (Figure 4A). The linker region between these two allows the OB domain to move and uncover the ATP-binding pocket, as seen in the crystal structure of the LIG-AMP intermediate. The OB domain moves ∼80^°^, which increases the space for DNA to bind and be positioned in the active site for repair (Figure 4C) (Unciuleac et al., 2019). We aligned the conserved catalytic core of the human NHEJ ligase LigIV (PDB 6BKG) (Kaminski et al., 2018), bound to a nicked DNA substrate, with the LIG-AMP intermediate structure (RMSD = 2.7 Å). While the fit is not perfect, the alignment shows the 5′-end of the DNA nick within the vicinity of the nucleotide binding pocket, and that opening of the OB domain during catalysis of ligation is likely necessary for active site access (Figure 4B). The movement of the OB domain is also predicted to facilitate formation of the ligase-AMP intermediate and release of the pyrophosphate. In the “closed” state, the OB domain stabilizes the γ-phosphate of ATP and when “opened”, the OB domain establishes contact with the backbone phosphates of the incoming DNA substrate (Akey et al., 2006; Unciuleac et al., 2019). While these interactions between the OB domain and ATP are not directly observed in either structure presented here, a structure of the LIG domain with ATP and MES suggests these interactions occur and that the interaction is dynamic (Unciuleac et al., 2019). Time-resolved x-ray crystallography may be a solution to acquire these intermediate conformations.

Capturing the active site in the pre-adenylylation state required mutating the catalytic lysine (K481) to methionine, to prevent formation of the LIG-AMP intermediate (Akey et al., 2006). A closer look at the pre-adenylylation state shows an ATP molecule, along with two magnesium ions in the active site (Figure 4D) (Akey et al., 2006). One magnesium ion is catalytic and is proposed to stabilize the transition state of the ATP by coordinating the ATP α-phosphate. This magnesium ion is also coordinated directly and indirectly through a water molecule by the conserved Glu613 (Akey et al., 2006). The second magnesium ion is thought to be non-catalytic, connecting with the β- and γ-phosphates of ATP. Amino acids surrounding the active site also stabilize the ATP molecule primarily through interactions with the α, β, and γ phosphates, and include amino acids His465, Arg501, Lys635, Lys637, and Arg629, while Arg486 forms a hydrogen bond with the ribose oxygen (Figure 4D) (Akey et al., 2006; Shuman and Glickman, 2007). It has been proposed that Lys635, Lys637 and the catalytic magnesium stabilize the adenylylation transition state, while the second metal ion works alongside three basic residues–Arg501, Arg629, and Lys635, to align the ATP γ-phosphate group for an in-line attack on ATP by the Lys481 nucleophile (Akey et al., 2006; Unciuleac et al., 2019). Mutational studies further confirmed the importance of these active site residues, where LigD with mutations Glu613Ala or Lys637Ala lacked nick-sealing activity (Akey et al., 2006), while LigD with mutations Arg501Ala or Arg629Ala had reduced nick-sealing activity compared to wild-type LigD (Unciuleac et al., 2019).

Once the adenylylation is complete and the LIG-AMP complex is formed, the active site residues still play a key role, but with some changes (Figure 4E) (Akey et al., 2006). A phosphoramidate bond is formed between the catalytic Lys481 and the phosphate group of AMP. This intermediate is coordinated in the active site through hydrogen bonds between a ribose sugar oxygen with Arg486 as in the pre-adenylylation state, while Arg501 shifts from the ATP γ-phosphate to the ribose sugar (Akey et al., 2006). A water-mediated interaction with Glu530 rounds out the hydrogen bonds to the ribose sugar. New hydrogen bonds also form in the active site between side-chains of Glu479 and Lys618, the backbone carbonyl of Gly480, and the backbone amide of Trp42 with the adenine base (Akey et al., 2006). These interactions highlight the substrate specificity of the active site for ATP.

From these crystal structures, some questions remain. Within the structure of the LIG-AMP intermediate, the electron density suggested the presence of a metal ion, although the question of whether one or both ions would fit in the active site is unclear. Additionally, LigD and the human homolog Ligase IV both preferentially ligate a nick containing a 3′ ribonucleotide (Zhu and Shuman, 2008; Pryor et al., 2018). While there are no structures of the LIG domain from LigD with DNA, Zhu and Shuman (2008) postulate that based on structures of *Escherichia coli* LigA, human LigI and *Chlorella* virus ligase bound to DNA nicks, the 3′-hydroxyl group is forced into an A-like conformation, similar to RNA. It is possible that bacterial LigD cannot restructure DNA into an A-form, thus requiring the presence of a 3′-ribonucleotide for optimal ligation (Zhu and Shuman, 2008). This theory is further supported by biochemical studies showing that the LIG domain alone was 12-fold faster for sealing a nick with a ribonucleotide compared to a nick with deoxyribonucleotides (Unciuleac et al., 2019). Future crystal structures of the LIG domain in complex with ribonucleotide-containing substrates will help solve this puzzle.

### The Polymerase Domain

The polymerase domain of LigD belongs to the archaeo-eukaryotic primase (AEP) superfamily (Iyer, 2005). The basic function of the POL domain of LigD is that of a nucleotidyltransferase, where a phosphodiester bond is created between an incoming nucleotide and the 3′-hydroxyl end of a DNA primer strand. At least 2 divalent metal ions are required, coordinated by conserved aspartate residues within the polymerase active site. One divalent metal ion primes the 3′-hydroxyl group of the primer strand for nucleophilic attack on the γ-phosphate of the incoming nucleotide, while the second divalent metal ion aids in the removal of the pyrophosphate, thus creating the phosphodiester bond between the primer strand, and incoming nucleotide (Beese and Steitz, 1991; Steitz, 1993; Steitz, 1999). The LigD POL domain is unique compared to other polymerases, containing a repertoire of nucleotidyltransferase functions not found in other members of the AEP superfamily, which typically function solely as primases during DNA replication (Pitcher et al., 2005; Guilliam et al., 2015). More specifically, the LigD POL domain is capable of DNA or RNA gap-filling, RNA primase, and terminal transferase activities (Weller et al., 2002; Della et al., 2004; Gong et al., 2004; Gong et al., 2005; Pitcher et al., 2005; Yakovleva and Shuman, 2006).

**FIGURE 5:**
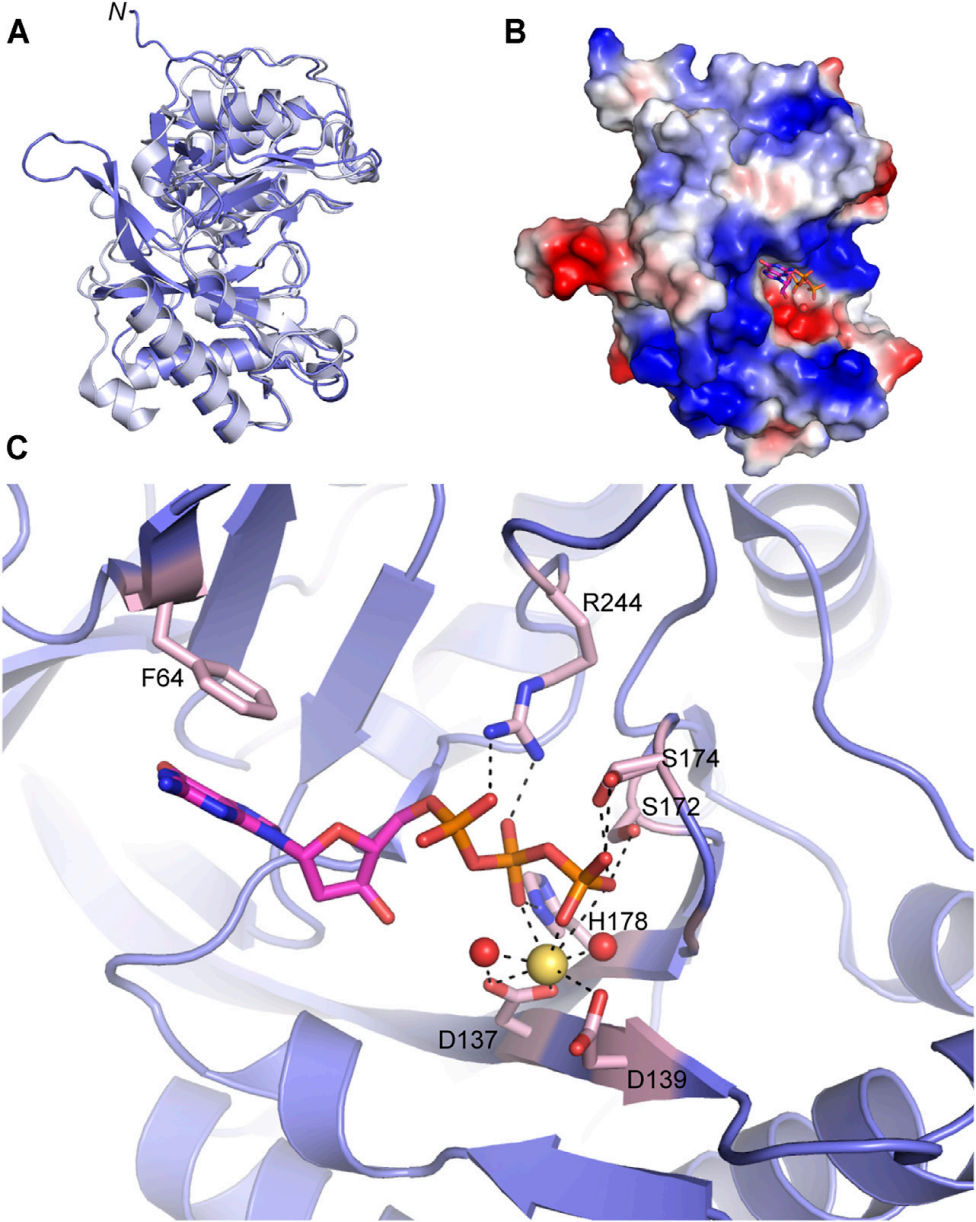
Atomic structure of the LigD polymerase domain. **(A)** Alignment of apo-LigD polymerase domain from *M. tuberculosis* (PDB 2IRU, purple) and *P. aeruginosa* (PDB 2FAO, grey), RMSD = 1.5 Å. **(B)** Electrostatic surface representation of *M. tuberculosis* LigD polymerase domain with dGTP and manganese in the active site (PDB 2IRY). Blue, positive charge; red, negative charge. **(C)** Active site of the polymerase domain from **(B)**. Key amino acids of the active site are colored with a pink backbone. dGTP, magenta sticks; manganese, yellow spheres; water molecules, red spheres; black dashed lines indicate hydrogen bonding. Figures generated with The PyMOL Molecular Graphics System, Version 2.0 Schrödinger, LLC.

As a member of the AEP superfamily, the LigD POL domain shares a common catalytic core, consisting of a N-terminal (αβ)_2_ unit that is packed onto a derived C-terminal RNA recognition motif (RRM) (Figure 5A) (Guilliam et al., 2015). Within this catalytic core lies three highly conserved motifs–an hhhDhD motif (where “h” is a hydrophobic residue, motif I), an sxH motif (where “s” is a small residue and “x” can be anything, motif II), and an hD/E motif (motif III) (Iyer, 2005; Guilliam et al., 2015). Motifs I and III are responsible for coordinating divalent metal ions during enzyme catalysis, whereas motif II binds the incoming nucleotide (Guilliam et al., 2015). Although the POL domain has considerable homology with replicative primases, its specialized role in DSB repair is attributed to its possession of unique structural elements in the regions following motif I, and in between motifs II and III (Koonin et al., 2000). These elements include a phosphate-binding pocket, in addition to a pair of distinct surface loops that aid with the synapsis of DNA breaks during NHEJ repair (Iyer, 2005).

Crystal structures of the POL domain from both *P. aeruginosa* and *M. tuberculosis* have been solved in the apo form (PDB 2FAO, 2IRU), and in the presence of nucleotides and divalent metal ions (PDB 2FAR, 2FAQ, 2IRY, and 2IRX) (Zhu et al., 2006; Pitcher et al., 2007b). The LigD POL domain apo structures from both species are well aligned (RMSD = 1.5 Å) (Figure 5A) and additional structures with DNA substrates have been solved from *M. tuberculosis*, therefore we will focus our attention on the *M. tuberculosis* structures.

The catalytic site of the POL domain is found in a cleft on the surface of the protein, surrounded by a positively charged region (Figure 5B), that functions both to align the incoming DNA substrate and mediate interactions between homodimers of the POL domain as will be discussed below (Brissett et al., 2007; Pitcher et al., 2007b; Brissett et al., 2013). In the presence of dGTP, the POL domain crystallized with a single divalent metal ion, manganese. Octahedral coordination of the manganese occurs with oxygen atoms of the conserved Asp137(motif I) and Asp139 (motif I) sidechains, oxygen atoms from the β- and γ-phosphates of dGTP and neighboring water molecules (Figure 5C) (Zhu et al., 2006; Pitcher et al., 2007b). The dGTP is further stabilized by a conserved triad of residues, Ser172 (motif II), His178 (motif II), and Arg244 with hydrogen bonds between the sidechains of the residues and oxygen atoms of the incoming nucleotide’s phosphate groups. A conserved Phe64 also base stacks with the guanosine ring (Pitcher et al., 2007b). Additional water molecules are involved in the hydrogen-bonding network that stabilizes the active site but have been left out of Figure 5C for clarity. Details of this hydrogen-bonding network are available in previous publications (Pitcher et al., 2007b).

**FIGURE 6:**
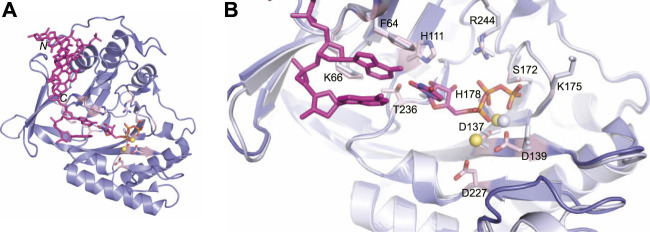
Atomic structure of the LigD polymerase domain in a pre-catalytic state. **(A)** Cartoon representation of the *M. tuberculosis* polymerase domain (PDB 3PKY) with a 3′-overhang DNA substrate (magenta), UTP with a magenta backbone and manganese as yellow spheres. Key amino acids of the active site are shown with a pink backbone. **(B)** Overlay of the active sites from the polymerase domain in the presence of ATP (PDB 2IRY, grey) and after the LIG-AMP intermediate has formed (PDB 3PKY, purple). DNA and the AMP intermediate are in magenta. Key amino acids and manganese ions relevant to the pre-adenylylation complex are in grey. Amino acids key to the active site after LIG-AMP formation have a pink backbone, while manganese ions are yellow spheres. Figures generated with The PyMOL Molecular Graphics System, Version 2.0 Schrödinger, LLC.

Missing from this structure was the presence of the second metal ion required for catalysis. However, crystallization of the LigD POL domain with UTP, manganese and DNA captured both metal ions in the active site (PDB 3PKY) (Brissett et al., 2011). The second metal ion forms a tetrahedral coordination with the same conserved aspartate residues, but also includes the side chain of an additional aspartate (Asp227, motif III) (Figure 6A). This interaction would prime the 3′-hydroxyl end of the DNA template strand, which is absent in this structure. The coordination of the manganese ion transforms the active site into a pre-catalytic complex, allowing UTP to base-pair with the adenine base of the templating DNA strand (Brissett et al., 2011). The conserved Phe64 base stacks with the templating DNA strand, rather than the incoming nucleotide as in Figure 5C (Figure 6B). New interactions in the active site further stabilize UTP, with hydrogen bonds forming between the 2′-hydroxyl group of the ribose, Thr236, and His111. An overlay of the active sites shows the changes of the amino acids in the active site when bound to dGTP vs the UTP:manganese:DNA complex. Of interest is that while a second divalent ion is observed in the tripartite complex, the triphosphate tail of dGTP likely occluded binding of the second manganese ion in the structure of the POL domain with dGTP (Brissett et al., 2011).

The ability of the LigD POL domain to insert ribonucleotides preferentially is of interest (Della et al., 2004; Pitcher et al., 2007a; Zhu and Shuman, 2010). Biochemical studies in *P. aeruginosa* show that the His111 homolog, when mutated in *P. aeruginosa* LigD, has reduced preference for ribonucleotides compared to wild-type LigD (Sánchez-Salvador and De Vega, 2020). In both tripartite complex structures from *M. tuberculosis* and *P. aeruginosa*, the conserved histidine hydrogen bonds to the 2′-hydroxyl of the ribose, dictating selectivity for ribonucleotides in the active site (Sánchez-Salvador and De Vega, 2020).The POL domain structure also plays a role in maintaining fidelity. *P. aeruginosa* LigD showed decreased polymerization efficiency and increased nucleotide misincorporation with a mutation of Lys606 to alanine (Sánchez-Salvador and De Vega, 2020). This lysine is conserved and in *M. tuberculosis*, the homologous Lys66 sandwiches the base of either the incoming nucleotide or templating DNA strand with Phe64 (Figure 6B) (Pitcher et al., 2007b; Brissett et al., 2011). While the exact mechanism of fidelity is unclear, it is hypothesized that the lysine helps select the correct nucleotide for insertion, when a DNA template is present (Sánchez-Salvador and De Vega, 2020).

**FIGURE 7:**
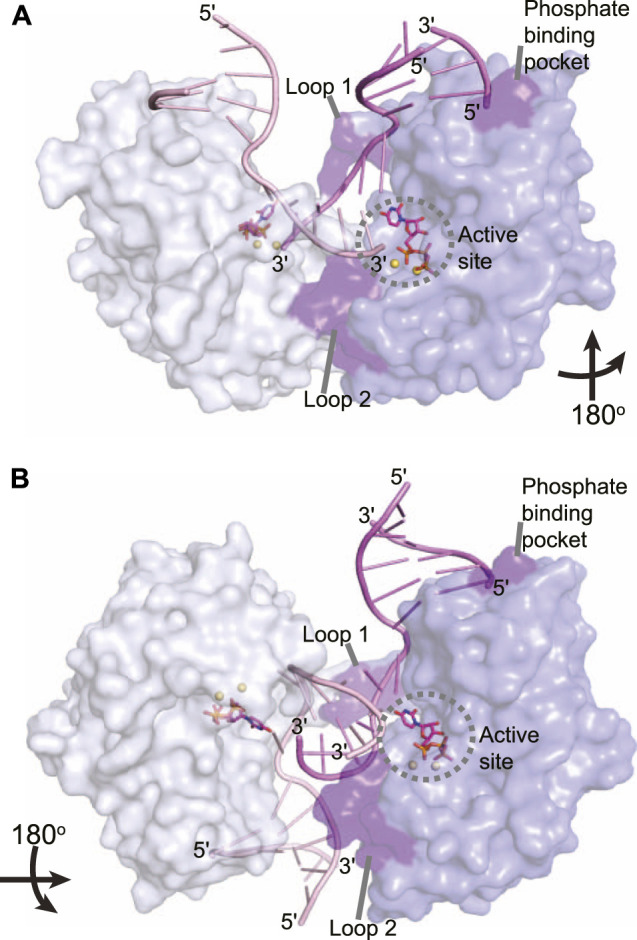
Atomic structures of the LigD polymerase domain from *M. tuberculosis* in complex with synapsed DNA substrates. **(A)** Surface representation of a chemically competent polymerase domain synaptic complex (PDB 4MKY). Each polymerase domain and associated dsDNA is individually colored. The pre-catalytic complex of the polymerase domain was aligned with the synaptic complex to indicate where the incoming nucleotide and metal ions would reside in relation to the DNA substrate (aligned with PDB 3PKY, RMSD = 0.37 Å). Key amino acids that stabilize the synaptic complex are highlighted in purple. **(B)** Surface representation of a chemically incompetent polymerase domain synaptic complex (PDB 2R9L). Each polymerase domain and associated dsDNA is individually colored. The pre-catalytic complex of the polymerase domain was aligned with the synaptic complex to indicate where the incoming nucleotide and metal ions would reside in relation to the DNA substrate (aligned with PDB 3PKY, RMSD = 0.34 Å). Key amino acids that stabilize the synaptic complex are highlighted in purple. Figures generated with The PyMOL Molecular Graphics System, Version 2.0 Schrödinger, LLC.

The LigD POL domain needs to be capable of handling complex DNA DSBs. While a gapped DNA substrate contains a connected template and primer strand, in the case of a DSB, the physical connection is severed, creating discontinuous template and primer strands. The mechanism by which LigD handles this challenge is to form a synaptic complex using two LigD proteins, as illustrated in crystal structures of the LigD POL domain in complex with DNA DSBs (Figure 7) (Brissett et al., 2007, 2013). We aligned the structure of the POL:UTP:DNA:Mn^2+^ with each synaptic complex (RMSD = 0.37 Å for Figure 7A, RMS = 0.34 Å for Figure 7B) to highlight the active site and relative location of the incoming nucleotide. How these POL domains interact with one another is dependent on whether the DNA ends are complementary or non-complementary. A catalytically competent synaptic arrangement is formed when bound to a DNA substrate with self-complementary ends (Figure 7A) (Brissett et al., 2013). Here, the POL domains are rotated 180^o^ around the y-axis with respect to one another, where the template strand from one POL domain becomes the incoming primer strand for the adjacent POL domain. Microhomologies between the 3′ ends of each incoming DNA strand helps stabilize the synapsis and provides the necessary primer strand that allows for an *in trans* polymerization mechanism. In essence, this configuration mimics gapped DNA substrates (Brissett et al., 2013). Critical to the formation of this complex is the phosphate binding pocket made up of Lys16, Lys26, and Asn13 on LigD, to bind a downstream 5′-phosphate group, where only one 5′-phosphate group is required for synapsis (Brissett et al., 2013). Conserved loops 1 and 2 of the POL domain also stabilize the synaptic complex (Brissett et al., 2007; Brissett et al., 2013), while loop 1 additionally interacts with the template strand to re-orient it to become a primer strand, while loop 2 guides the 3′ DNA end into the polymerase active site (Brissett et al., 2013). In contrast, when the POL domain is bound to a non-complementary DNA substrate, a synapse is also created, but one that is catalytically incompetent for polymerization extension (Figure 7B). In this structure, the POL domains are rotated 180^o^ around the x-axis with respect to each other, with the 3′-DNA ends of opposing DNA substrates forming a synaptic complex (Brissett et al., 2007). The 5′-phosphate of the downstream DNA strand is still bound by the phosphate-biding pocket, and loop 1 is critical to forming the synaptic complex and re-orienting the incoming 3′-end, with loop 2 also contacting the DNA (Brissett et al., 2007). However, because the DNA ends are not complementary, the DNA is distorted, with only small microhomologies being formed. These distortions result in the 3′ ends failing to reach the catalytic site for extension, instead forming hairpin structures. It is hypothesized that the space between the synapsed POL domains could accommodate the PE domain and allow additional processing of the DNA ends prior to polymerization (Brissett et al., 2007).

### The Phosphoesterase Domain

When first discovered, the PE domain lacked significant homology to any known family of bacterial nucleases, and thus was classified as a new phosphoesterase family (Zhu and Shuman, 2005b). Since then, this phosphoesterase domain has also been found in proteomes of both archaea and eukaryotes (Nair et al., 2010). The PE domain is multifunctional–reflective of its role in a multipurpose enzyme. It is a phosphoesterase, converting 3′-phosphate ends to the necessary 3′-hydroxyl for ligation, while also being a ribonuclease, resecting lengths of 3′-ribonucleotides introduced by the POL domain, and leaving the preferred single 3′-ribonucleotide for ligation (Zhu and Shuman, 2005b, 2008). The mechanism by which the PE domain carries out 3′ end-healing activities occurs in two manganese-dependent steps (Zhu and Shuman, 2005b). First, removal of a 3′-terminal nucleoside on the primer strand leaves behind a ribonucleoside with a 3′-phosphate group (phosphodiesterase activity). Then, via hydrolysis, the 3′-phosphate group is converted to a 3′-hydroxyl group with the release of an inorganic phosphate (phosphomonoesterase activity) (Zhu and Shuman, 2005b; Zhu and Shuman, 2006; Zhu et al., 2005; Nair et al., 2010). These activities create a comprehensive 3′ end-processing mechanism in the PE domain necessary for DSB repair and is found in *P. aeruginosa, A. tumefaciens,* and mycobacterial LigD (Zhu and Shuman, 2005b; Zhu and Shuman, 2006; Zhu and Shuman, 2007).

**FIGURE 8:**
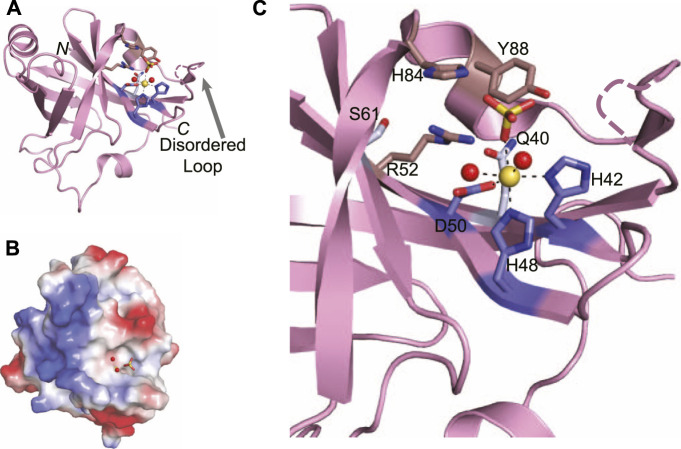
Atomic structure of the LigD phosphoesterase domain from *P. aeruginosa* (PDB 3N9B). **(A)** Cartoon representation of the phosphoesterase domain with a sulfate ion (yellow sticks), manganese ion (yellow sphere), and water molecules (red spheres) in the active site. Amino acids critical to the active site are shown as sticks. Amino acids with a purple backbone interact with the manganese ion. Amino acids with a brown backbone interact with the sulfate ion and amino acids with a grey backbone stabilize the active site structure. Black dashed lines indicate hydrogen bonding. Pink dashed lines represent a disordered loop that was absent from the structure. **(B)** Electrostatic surface representation of the phosphoesterase domain. Blue, positive charge; red, negative charge. **(C)** Close-up of the active site of the phosphoesterase domain, colored as in **(A)**. Figures generated with The PyMOL Molecular Graphics System, Version 2.0 Schrödinger, LLC.

Atomic structures of the *P. aeruginosa* LigD PE domain have been solved by both x-ray crystallography and nuclear magnetic resonance (PDB 3N9B, 2LJ6) (Nair et al., 2010; Natarajan et al., 2012). Additional structures of archaeal PE domains from *Candidatus Korarchaeum cryptofilum* and *Methanosarcina barkeri* are more compact versions of *P. aeruginosa* PE domain, although the catalytic core is conserved (Nair et al., 2010). The *Methanocella paludicola* PE domain (PDB 5DMP) (Bartlett et al., 2016), though, aligns well with the *P. aeruginosa* PE domain (RMSD = 0.45 Å), therefore we will focus the remainder of our discussion on the *P. aeruginosa* PE domain. The PE domain forms an eight-stranded beta barrel, that is bounded by two alpha helices and a 3_10_ helix (Figure 8A) (Nair et al., 2010). The beta barrel structure is maintained through a cluster of conserved hydrophobic residue pairs, while the hydrophilic active site is situated on the exterior of the barrel, in a crescent-shaped groove (Figure 8C) (Nair et al., 2010). Alongside the active site runs a stretch of positive charge (Figure 8B), which could interact with the negatively charged backbone of DNA, guiding the 3′-DNA end into the active site for processing.

Within the structure of the active site are the required manganese ion and a sulfate anion that can be considered a mimetic for the scissile phosphate to be cleaved in the DNA backbone (Nair et al., 2010). The manganese is stabilized by an octahedral coordination complex, mediated by a catalytic triad of conserved residues–His42, His48, and Asp50–that when expressed as alanine point mutations in *P. aeruginosa* LigD, are catalytically dead (Zhu and Shuman, 2006). Two neighboring water molecules and the oxygen from the sulfate anion complete the coordination of the manganese ion (Nair et al., 2010). The sulfate anion is proposed to stabilize the transition state and is coordinated by another highly conserved group of residues, Arg52, His84, and Tyr88. Like the catalytic triad surrounding the manganese, point mutations of His84, and Tyr88 in *P.aeruginosa* LigD are also catalytically inactive, highlighting the necessity of these residues in the phosphoesterase active site (Zhu and Shuman, 2006). Also within the active site are two more conserved residues, Gln40, and Ser 61. While these residues do not appear necessary for the catalytic mechanism, they may be key to forming the structure of the active site, along with Arg52, as LigD proteins with a Gln40Ala mutation had severely reduced phosphodiesterase activity compared to wild-type LigD (Zhu and Shuman, 2006; Nair et al., 2010).

Biochemical studies have also identified that Arg14, Arg15, Glu21, and Glu82 are necessary for 3′-phosphatase activity, but not removal of the 3′-ribonucleoside (Zhu and Shuman, 2006). The N-terminus of the PE domain is disordered in structures from both x-ray crystallography and nuclear magnetic resonance (Nair et al., 2010; Natarajan et al., 2012), precluding structural information about Arg14, Arg15, and Glu21. However, Glu82 resides on the outer edge of the active site. While in the current structure of the PE domain, the side chain of Glu82 is pointed away from the active site, it is likely that in the presence of the DNA substrate, Glu82 may become more involved, given its relevance in the biochemical activity of the PE domain.

Absent in the crystal structure of the PE domain is a loop outside the active site (Figure 8A, dashed line), which is seen in crystal structures of the archaeal PE domain from *Methanocella paludicola* (Bartlett et al., 2016). Based on a lack of density for this loop in the *P. aeruginosa* PE domain structure (Nair et al., 2010), coupled with evidence from nuclear magnetic resonance studies showing that this loop moves away from the active site when DNA is present (Natarajan et al., 2012), it is likely this loop is flexible. In the *M. paludicola* PE domain, the loop appears to cover the active site, which contains a magnesium and vanadate ion (Bartlett et al., 2016). A similar arrangement is likely for the structure of the PE domain in *P. aeruginosa,* given the current trajectory of the ends of the loop that are visible, and the presence of similar substrates in the active site. Future structures of this domain with DNA may well show that this loop acts as a capping mechanism, allowing access to the active site only in the presence of the correct DNA substrate.

### Coordinating Repair in LigD

Atomic structures of the LIG, POL, and PE domain from bacteria and archaea, combined with biochemical studies, continue to be instrumental in highlighting the structural foundation for enzymatic activity in LigD. However, the arrangement of these domains in three-dimensional space in wild-type LigD is unknown, along with how the structural arrangement affects processing of the DNA DSB. This idea is especially interesting, given that the primary structure of the domains can vary between bacterial species (Figure 3). Until an experimental structure is obtained, either by cryo-electron microscopy or x-ray crystallography, recent advances in *in silico* structure predictions using the AlphaFold algorithm (Jumper et al., 2021) through the ColabFold notebook (Mirdita et al., 2021), provide an opportunity to explore possible models of wild-type LigD.

**FIGURE 9:**
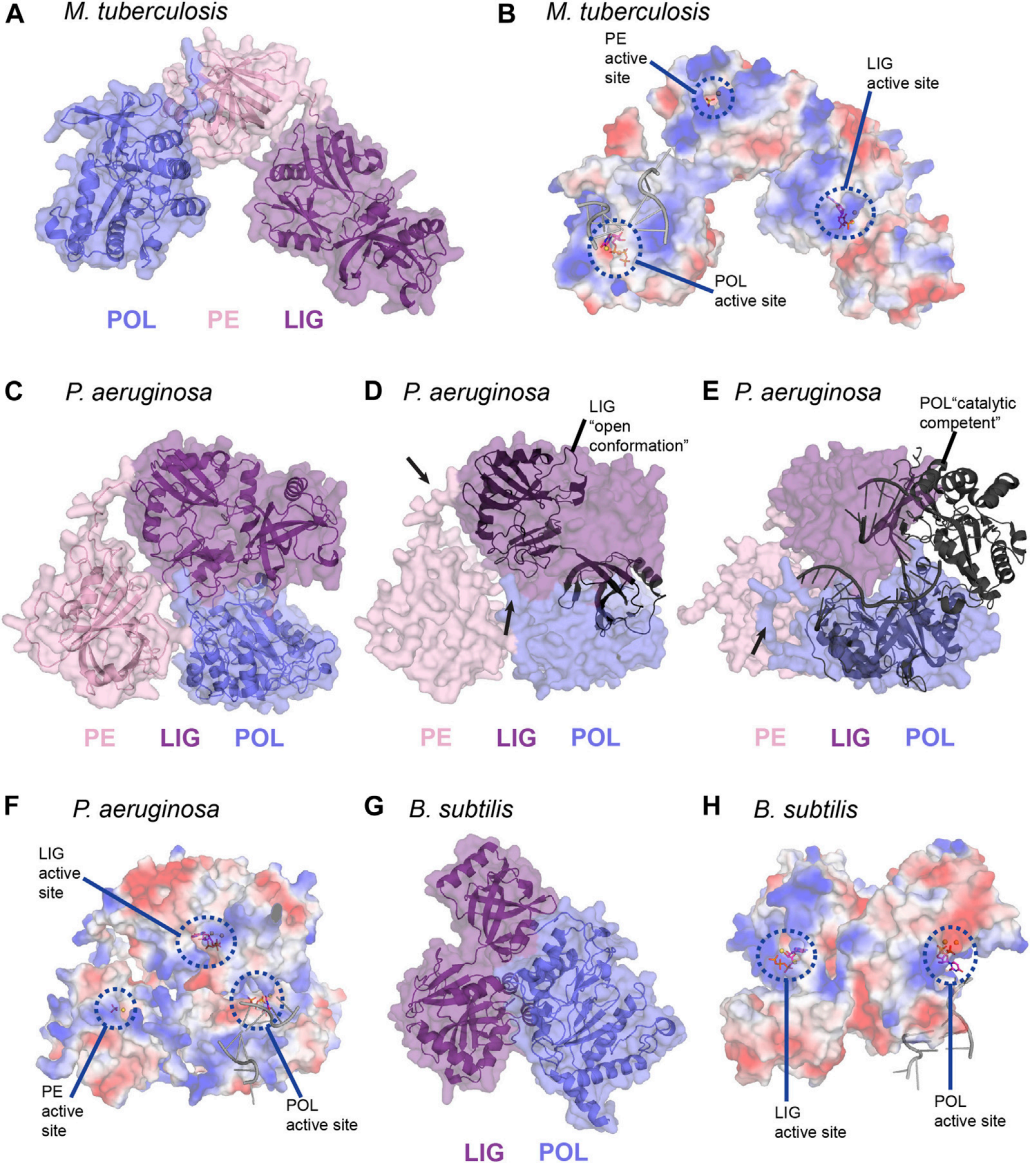
*In silico* predictions of LigD atomic structure **(A)** *M. tuberculosis* LigD, predicted by AlphaFold (Jumper et al., 2021), displayed as both cartoon and surface representations. POL–blue; PE–pink; LIG–purple **(B)** Electrostatic surface representation of **(A)**, aligned with the NTase fold of the ligase domain from PDB 6NHZ, the pre-catalytic polymerase domain from PDB 3PKY, and the phosphoesterase domain from PDB 3N9B. Active sites of each enzymatic domain are encircled by blue dashes. Electropositive–blue; Electronegative–red **(C)** *P. aeruginosa* LigD, predicted by ColabFold (Mirdita et al., 2021), displayed as both cartoon and surface representations. POL–blue; PE–pink; LIG–purple **(D)** Surface representation of *P. aeruginosa* LigD model aligned with the open conformation of the ligase domain from *M. tuberculosis* shown as a cartoon (PDB 1VS0, RMSD = 1.1 Å). Black arrows represent locations of potential flexible loops **(E)** Surface representation of *P. aeruginosa* LigD model aligned with the catalytically competent polymerase domain synaptic complex from *M. tuberculosis*, shown as a cartoon (PDB 4MKY, RMSD = 1.4 Å). Black arrows represent locations of potential flexible loops **(F)** Electrostatic surface representation of **(C)**, aligned with the NTase fold of the ligase domain from PDB 6NHZ, the pre-catalytic polymerase domain from PDB 3PKY and the phosphoesterase domain from PDB 3N9B. Active sites of each enzymatic domain are encircled by blue dashes. Electropositive–blue; Electronegative–red **(G)** *B. subtilis* LigD, predicted by ColabFold (Mirdita et al., 2021), displayed as both cartoon and surface representations. POL–blue; LIG–purple **(H)** Electrostatic surface representation of **(G)**, aligned with the NTase fold of the ligase domain from PDB 6NHZ and the pre-catalytic polymerase domain from PDB 3PKY. Active sites of each enzymatic domain are encircled by blue dashes. Electropositive–blue; Electronegative–red. Figures generated with The PyMOL Molecular Graphics System, Version 2.0 Schrödinger, LLC.

We generated predictions of LigD from *P. aeruginosa,* and *B. subtilis* using the ColabFold notebook, and downloaded the prediction of *M. tuberculosis* LigD from the AlphaFold Protein Structure Database (Figure 9) (Jumper et al., 2021; Mirdita et al., 2021). Interestingly, both *P. aeruginosa,* and *B. subtilis* LigD have compact conformations, with the enzymatic domains arranging into an almost globular structure. *M. tuberculosis* though, is slightly more elongated, forming a curved, horseshoe like structure (Figures 9A,C,G). Are these compact folds functionally active in the LigD models? The LigD LIG domain contains an NTase and OB domain, where the OB domain is flexible, and existing in both open and closed conformations as described earlier. We overlaid the structure of the *M. tuberculosis* LIG domain in the open conformation, using the NTase domain for alignment (PDB 1VS0) (Akey et al., 2006) with each LigD model. We found that for *M. tuberculosis* and *B. subtilis* LigD models, the OB fold is in between the open and closed conformation, however for the *P. aeruginosa* LigD model, the OB fold is closed, capping the active site. Interestingly, if the OB fold were to open, it would collide with the POL domain in the current model (Figure 9D). Therefore, some flexibility must be inherent in these structures, at the least in *P. aeruginosa* LigD to accommodate accessibility of the active site. Flexibility is also essential if the structure of the POL domain bound to a synaptic DNA substrate (PDB 4MKY) (Brissett et al., 2013) is considered as well. Homodimerization of LigD, via the POL domain bound to a DNA synapse would not cause any conflicts in *M. tuberculosis* or *B. subtilis* LigD, however in the present model of *P. aeruginosa,* the POL domain would collide with the LIG domain (Figure 9E). Long loops predicted between the POL, LIG, and PE domains are likely to be flexible, based on the lower confidence scores in positioning these loops by ColabFold and would permit alternate conformations of *P. aeruginosa* LigD. Small-angle x-ray scattering studies may be able to answer these questions about the movement of LigD in solution.

We also aligned structures of the LIG, POL, and PE domains containing substrates in their active site, with the LigD models to better visualize how repair may occur (Figures 9B,F,H). For the LIG domain, we used the substrates of ATP and magnesium (PDB 6NHZ) (Unciuleac et al., 2019), creating an alignment through the NTase fold, while for the POL domain, we used the complex containing DNA, UTP, and manganese (PDB 3PKY) (Brissett et al., 2011). For the PE domain, we used the substrates of manganese and the sulfate ion (PDB 3N9B) (Nair et al., 2010) in the alignment. As shown in the electrostatic surface models (Figures 9B,F,H), the active sites of each domain are on the outer surface and accessible to the solvent, with the exception of the trapped LIG active site in *P. aeruginosa*, and in the absence of a synaptic complex in the *P. aeruginosa* POL domain. The electrostatic surface shows trails of positive regions leading to the active sites and may form a guiding path for the DNA ends in need of polymerase extension, ribonucleoside removal or ligation of the DSB. This path would allow a DNA DSB to move between the different enzymatic domains, depending on the processing requirements at the DSB, with active site structures allowing or denying access to the DSB, depending on whether the DNA substrate is a fit. Alternately, LigD may move around the DSB, capturing and releasing the ends from the active site of each domain, carrying out repair. Ku may also have a role in coordinating LigD-DNA binding. *M. tuberculosis* Ku binds the POL domain of LigD and weakly to the LIG domain, which could change the conformation of LigD to better accommodate DNA ends (Della et al., 2004; Pitcher et al., 2005; Wright et al., 2010). Answers to these questions and more are to be found in future experimental structures of LigD, bound to Ku and/or in the midst of repairing a DNA DSB.

## Concluding Remarks

Over the past 20 years, structures of the individual LigD domains combined with elegant biochemical and genetic studies have made vast inroads on the way to understanding the details of NHEJ repair, yet the structure of the wild-type LigD on its own, or with its repair partner Ku, has remained elusive. The newly accessible AlphaFold algorithm, combined with the ColabFold notebook (Jumper et al., 2021; Mirdita et al., 2021) has provided a means to examine models of wild-type LigD. Studying these models in combination with experimentally determined LigD structures can generate new research avenues that will continue to unravel the molecular mechanism of NHEJ repair. NHEJ exists in several pathogenic bacteria, such as *M. tuberculosis*, therefore a better understanding of the proteins involved in this pathway may provide insight into antibiotic tolerance and could lead to new targets for antibacterial therapies. Also, discoveries in bacterial NHEJ may lead to the identification of new features in eukaryotic NHEJ, which could be of benefit to cancer therapeutics.

## References

[B1] Akey D. Martins A. Aniukwu J. Glickman M. S. Shuman S. Berger J. M. (2006). Crystal Structure and Nonhomologous End-Joining Function of the Ligase Component of Mycobacterium DNA Ligase D. J. Biol. Chem. 281, 13412–13423. 10.1074/jbc.M513550200 16476729

[B2] Andres S. N. Schellenberg M. J. Wallace B. D. Tumbale P. Williams R. S. (2015). Recognition and Repair of Chemically Heterogeneous Structures at DNA Ends. Environ. Mol. Mutagen. 56, 1–21. 10.1002/em.21892 25111769 PMC4303525

[B3] Aravind L. Koonin E. V. (2001). Prokaryotic Homologs of the Eukaryotic DNA-End-Binding Protein Ku, Novel Domains in the Ku Protein and Prediction of a Prokaryotic Double-Strand Break Repair System. Genome Res. 11, 1365–1374. 10.1101/gr.181001 11483577 PMC311082

[B4] Ayora S. Carrasco B. Cárdenas P. P. César C. E. Cañas C. Yadav T. (2011). Double-strand Break Repair in Bacteria: a View fromBacillus Subtilis. FEMS Microbiol. Rev. 35, 1055–1081. 10.1111/J.1574-6976.2011.00272.X 21517913

[B5] Bartlett E. J. Brissett N. C. Plocinski P. Carlberg T. Doherty A. J. (2016). Molecular Basis for DNA Strand Displacement by NHEJ Repair Polymerases. Nucleic Acids Res. 44, 2173–2186. 10.1093/nar/gkv965 26405198 PMC4797286

[B6] Beese L. S. Steitz T. A. (1991). Structural Basis for the 3′-5′ Exonuclease Activity of *Escherichia coli* DNA Polymerase I: a Two Metal Ion Mechanism. EMBO J. 10, 25–33. 10.1002/J.1460-2075.1991.TB07917.X 1989886 PMC452607

[B7] Bowater R. Doherty A. J. Botchan M. Stringer J. Mitchison T. Sambrook J. (2006). Making Ends Meet: Repairing Breaks in Bacterial DNA by Non-homologous End-Joining. Plos Genet. 2, e8. 10.1371/journal.pgen.0020008 16518468 PMC1378119

[B8] Brandsma I. Gent D. C. (2012). Pathway Choice in DNA Double Strand Break Repair: Observations of a Balancing Act. Genome Integr. 3, 9–10. 10.1186/2041-9414-3-9 23181949 PMC3557175

[B9] Brissett N. C. Martin M. J. Bartlett E. J. Bianchi J. Blanco L. Doherty A. J. (2013). Molecular Basis for DNA Double-Strand Break Annealing and Primer Extension by an NHEJ DNA Polymerase. Cell Rep. 5, 1108–1120. 10.1016/j.celrep.2013.10.016 24239356 PMC3898472

[B10] Brissett N. C. Martin M. J. Pitcher R. S. Bianchi J. Juarez R. Green A. J. (2011). Structure of a Preternary Complex Involving a Prokaryotic NHEJ DNA Polymerase. Mol. Cell 41, 221–231. 10.1016/j.molcel.2010.12.026 21255731

[B11] Brissett N. C. Pitcher R. S. Juarez R. Picher A. J. Green A. J. Dafforn T. R. (2007). Structure of a NHEJ Polymerase-Mediated DNA Synaptic Complex. Science 318, 456–459. 10.1126/science.1145112 17947582

[B12] Brzostek A. Szulc I. Klink M. Brzezinska M. Sulowska Z. Dziadek J. (2014). Either Non-Homologous Ends Joining or Homologous Recombination Is Required to Repair Double-Strand Breaks in the Genome of Macrophage-Internalized *Mycobacterium tuberculosis* . PLoS One 9, e92799. 10.1371/journal.pone.0092799 24658131 PMC3962454

[B13] de Vega M. (2013). The Minimal Bacillus Subtilis Nonhomologous End Joining Repair Machinery. PLoS One 8, e64232. 10.1371/journal.pone.0064232 23691176 PMC3656841

[B14] Della M. Palmbos P. L. Tseng H.-M. Tonkin L. M. Daley J. M. Topper L. M. (2004). Mycobacterial Ku and Ligase Proteins Constitute a Two-Component NHEJ Repair Machine. Science 306, 683–685. 10.1126/science.1099824 15499016

[B15] de Ory A. Zafra O. de Vega M. (2014). Efficient Processing of Abasic Sites by Bacterial Nonhomologous End-Joining Ku Proteins. Nucleic Acids Res. 42, 13082–13095. 10.1093/nar/gku1029 25355514 PMC4245934

[B16] Gong C. Bongiorno P. Martins A. Stephanou N. C. Zhu H. Shuman S. (2005). Mechanism of Nonhomologous End-Joining in Mycobacteria: A Low-Fidelity Repair System Driven by Ku, Ligase D and Ligase C. Nat. Struct. Mol. Biol. 12, 304–312. 10.1038/nsmb915 15778718

[B17] Gong C. Martins A. Bongiorno P. Glickman M. Shuman S. (2004). Biochemical and Genetic Analysis of the Four DNA Ligases of Mycobacteria. J. Biol. Chem. 279, 20594–20606. 10.1074/jbc.M401841200 14985346

[B18] Guilliam T. A. Keen B. A. Brissett N. C. Doherty A. J. (2015). Primase-polymerases Are a Functionally Diverse Superfamily of Replication and Repair Enzymes. Nucleic Acids Res. 43, 6651–6664. 10.1093/nar/gkv625 26109351 PMC4538821

[B19] Iyer L. M. (2005). Origin and Evolution of the Archaeo-Eukaryotic Primase Superfamily and Related palm-domain Proteins: Structural Insights and New Members. Nucleic Acids Res. 33, 3875–3896. 10.1093/nar/gki702 16027112 PMC1176014

[B20] Jumper J. Evans R. Pritzel A. Green T. Figurnov M. Ronneberger O. (20212021). Highly Accurate Protein Structure Prediction with AlphaFold. Nature 596, 583–589. 10.1038/s41586-021-03819-2 PMC8371605 34265844

[B21] Kaminski A. M. Tumbale P. P. Schellenberg M. J. Williams R. S. Williams J. G. Kunkel T. A. (2018). Structures of DNA-Bound Human Ligase IV Catalytic Core Reveal Insights into Substrate Binding and Catalysis. Nat. Commun. 9, 2642. 10.1038/s41467-018-05024-8 29980672 PMC6035275

[B22] Koonin E. V. Wolf Y. I. Kondrashov A. S. Aravind L. (2000). Bacterial Homologs of the Small Subunit of Eukaryotic DNA Primase. J. Mol. Microbiol. Biotechnol. 2, 509–512. 10.1159/issn.1660-2412 11075926

[B23] Korycka-Machala M. Brzostek A. Rozalska S. Rumijowska-Galewicz A. Dziedzic R. Bowater R. (2006). Distinct DNA Repair Pathways Involving RecA and Nonhomologous End Joining in Mycobacterium Smegmatis. FEMS Microbiol. Lett. 258, 83–91. 10.1111/J.1574-6968.2006.00199.X 16630260

[B24] Kowalczykowski S. C. Dixon D. A. Eggleston A. K. Lauder S. D. Rehrauer W. M. (1994). Biochemistry of Homologous Recombination in *Escherichia coli* . Microbiol. Rev. 58 (3), 401–465. 7968921 10.1128/mr.58.3.401-465.1994 PMC372975

[B25] Kushwaha A. K. Grove A. (2013a). C-terminal Low-Complexity Sequence Repeats of *Mycobacterium Smegmatis* Ku Modulate DNA Binding. Biosci. Rep. 33, e00016. 10.1042/BSR20120105 23167261 PMC3553676

[B26] Kushwaha A. K. Grove A. (2013b). Mycobacterium Smegmatis Ku Binds DNA without Free Ends. Biochem. J. 456, 275–282. 10.1042/BJ20130749 24059867

[B27] Leggett M. J. Mcdonnell G. Denyer S. P. Setlow P. Maillard J.-Y. (2012). Bacterial Spore Structures and Their Protective Role in Biocide Resistance. J. Appl. Microbiol. 113, 485–498. 10.1111/j.1365-2672.2012.05336.x 22574673

[B28] Li X. Heyer W.-D. (2008). Homologous Recombination in DNA Repair and DNA Damage Tolerance. Cell Res 18, 99–113. 10.1038/cr.2008.1 18166982 PMC3087377

[B29] McGovern S. Baconnais S. Roblin P. Nicolas P. Drevet P. Simonson H. (2016). C-terminal Region of Bacterial Ku Controls DNA Bridging, DNA Threading and Recruitment of DNA Ligase D for Double Strand Breaks Repair. Nucleic Acids Res. 44, 4785–4806. 10.1093/nar/gkw149 26961308 PMC4889933

[B30] Mirdita M. Ovchinnikov S. Steinegger M. (2021). ColabFold - Making Protein Folding Accessible to All. bioRxiv. 10.1101/2021.08.15.456425 PMC9184281 35637307

[B31] Moeller R. Stackebrandt E. Reitz G. Berger T. Rettberg P. Doherty A. J. (2007). Role of DNA Repair by Nonhomologous-End Joining in Bacillus Subtilis Spore Resistance to Extreme Dryness, Mono- and Polychromatic UV, and Ionizing Radiation. J. Bacteriol. 189, 3306–3311. 10.1128/JB.00018-07 17293412 PMC1855867

[B32] Nair P. A. Smith P. Shuman S. (2010). Structure of Bacterial LigD 3'-phosphoesterase Unveils a DNA Repair Superfamily. Proc. Natl. Acad. Sci. 107, 12822–12827. 10.1073/pnas.1005830107 20616014 PMC2919965

[B33] Natarajan A. Dutta K. Temel D. B. Nair P. A. Shuman S. Ghose R. (2012). Solution Structure and DNA-Binding Properties of the Phosphoesterase Domain of DNA Ligase D. Nucleic Acids Res. 40, 2076–2088. 10.1093/nar/gkr950 22084199 PMC3300020

[B34] Oates M. E. Romero P. Ishida T. Ghalwash M. Mizianty M. J. Xue B. (2013). D2P2: Database of Disordered Protein Predictions. Nucleic Acids Res. 41, D508–D516. 10.1093/nar/gks1226 23203878 PMC3531159

[B35] Öz R. Wang J. L. Guerois R. Goyal G. Kk S. Ropars V. (2021). Dynamics of Ku and Bacterial Non-homologous End-Joining Characterized Using Single DNA Molecule Analysis. Nucleic Acids Res. 49, 2629–2641. 10.1093/nar/gkab083 33590005 PMC7969030

[B36] Palomino J. Martin A. (2014). Drug Resistance Mechanisms in *Mycobacterium tuberculosis* . Antibiotics 3, 317–340. 10.3390/antibiotics3030317 27025748 PMC4790366

[B37] Pitcher R. S. Brissett N. C. Doherty A. J. (2007a). Nonhomologous End-Joining in Bacteria: A Microbial Perspective. Annu. Rev. Microbiol. 61, 259–282. 10.1146/annurev.micro.61.080706.093354 17506672

[B38] Pitcher R. S. Brissett N. C. Picher A. J. Andrade P. Juarez R. Thompson D. (2007b). Structure and Function of a Mycobacterial NHEJ DNA Repair Polymerase. J. Mol. Biol. 366, 391–405. 10.1016/j.jmb.2006.10.046 17174332

[B39] Pitcher R. S. Green A. J. Brzostek A. Korycka-Machala M. Dziadek J. Doherty A. J. (2007c). NHEJ Protects Mycobacteria in Stationary Phase against the Harmful Effects of Desiccation. DNA Repair 6, 1271–1276. 10.1016/J.DNAREP.2007.02.009 17360246

[B40] Pitcher R. S. Tonkin L. M. Green A. J. Doherty A. J. (2005). Domain Structure of a NHEJ DNA Repair Ligase from *Mycobacterium tuberculosis* . J. Mol. Biol. 351, 531–544. 10.1016/J.JMB.2005.06.038 16023671

[B42] Pryor J. M. Conlin M. P. Carvajal-Garcia J. Luedeman M. E. Luthman A. J. Small G. W. (2018). Ribonucleotide Incorporation Enables Repair of Chromosome Breaks by Nonhomologous End Joining. Science 361, 1126–1129. 10.1126/SCIENCE.AAT2477 30213916 PMC6252249

[B43] Sánchez-Salvador A. De Vega M. (2020). Structural Determinants Responsible for the Preferential Insertion of Ribonucleotides by Bacterial NHEJ PolDom. Biomolecules 10, 203. 10.3390/biom10020203 PMC7072297 32019147

[B44] Shuman S. Glickman M. S. (2007). Bacterial DNA Repair by Non-homologous End Joining. Nat. Rev. Microbiol. 5, 852–861. 10.1038/nrmicro1768 17938628

[B45] Steitz T. A. (1999). DNA Polymerases: Structural Diversity and Common Mechanisms. J. Biol. Chem. 274, 17395–17398. 10.1074/JBC.274.25.17395 10364165

[B46] Steitz T. A. (1993). DNA- and RNA-dependent DNA Polymerases. Curr. Opin. Struct. Biol. 3, 31–38. 10.1016/0959-440X(93)90198-T

[B47] Stephanou N. C. Gao F. Bongiorno P. Ehrt S. Schnappinger D. Shuman S. (2007). Mycobacterial Nonhomologous End Joining Mediates Mutagenic Repair of Chromosomal Double-Strand DNA Breaks. J. Bacteriol. 189, 5237–5246. 10.1128/JB.00332-07 17496093 PMC1951864

[B48] Tomkinson A. E. Vijayakumar S. Pascal J. M. Ellenberger T. (2006). DNA Ligases: Structure, Reaction Mechanism, and Function. Chem. Rev. 106, 687–699. 10.1021/cr040498d 16464020

[B49] Unciuleac M.-C. Goldgur Y. Shuman S. (2019). Structures of ATP-Bound DNA Ligase D in a Closed Domain Conformation Reveal a Network of Amino Acid and Metal Contacts to the ATP Phosphates. J. Biol. Chem. 294, 5094–5104. 10.1074/jbc.RA119.007445 30718283 PMC6442053

[B50] Weller G. R. Doherty A. J. (2001). A Family of DNA Repair Ligases in Bacteria? FEBS Lett. 505, 340–342. 10.1016/S0014-5793(01)02831-9 11566200

[B51] Weller G. R. Kysela B. Roy R. Tonkin L. M. Scanlan E. Della M. (2002). Identification of a DNA Nonhomologous End-Joining Complex in Bacteria. Science 297, 1686–1689. 10.1126/science.1074584 12215643

[B52] Wright D. DeBeaux A. Shi R. Doherty A. J. Harrison L. (2010). Characterization of the Roles of the Catalytic Domains of *Mycobacterium tuberculosis* Ligase D in Ku-dependent Error-Prone DNA End Joining. Mutagenesis 25, 473–481. 10.1093/MUTAGE/GEQ029 20530153 PMC2925156

[B53] Wright W. D. Shah S. S. Heyer W.-D. (2018). Homologous Recombination and the Repair of DNA Double-Strand Breaks. J. Biol. Chem. 293, 10524–10535. 10.1074/JBC.TM118.000372 29599286 PMC6036207

[B54] Zhao B. Rothenberg E. Ramsden D. A. Lieber M. R. (2020). The Molecular Basis and Disease Relevance of Non-homologous DNA End Joining. Nat. Rev. Mol. Cell Biol. 21, 765–781. 10.1038/s41580-020-00297-8 33077885 PMC8063501

[B41] Yakovleva L. Shuman S. (2006). Nucleotide Misincorporation, 3′-Mismatch Extension, and Responses to Abasic Sites and DNA Adducts by the Polymerase Component of Bacterial DNA Ligase D. J. Biol. Chem. 281, 25026–25040. 10.1074/jbc.M603302200 16816388

[B55] Zhu H. Nandakumar J. Aniukwu J. Wang L. K. Glickman M. S. Lima C. D. (2006). Atomic Structure and Nonhomologous End-Joining Function of the Polymerase Component of Bacterial DNA Ligase D. Proc. Natl. Acad. Sci. 103, 1711–1716. 10.1073/pnas.0509083103 16446439 PMC1413644

[B56] Zhu H. Shuman S. (2005a). A Primer-dependent Polymerase Function of *Pseudomonas aeruginosa* ATP-dependent DNA Ligase (LigD). J. Biol. Chem. 280, 418–427. 10.1074/jbc.M410110200 15520014

[B57] Zhu H. Shuman S. (2008). Bacterial Nonhomologous End Joining Ligases Preferentially Seal Breaks with a 3′-OH Monoribonucleotide. J. Biol. Chem. 283, 8331–8339. 10.1074/jbc.M705476200 18203718 PMC2276377

[B58] Zhu H. Shuman S. (2007). Characterization of Agrobacterium Tumefaciens DNA Ligases C and D. Nucleic Acids Res. 35, 3631–3645. 10.1093/nar/gkm145 17488851 PMC1920237

[B59] Zhu H. Shuman S. (2010). Gap Filling Activities of Pseudomonas DNA Ligase D (LigD) Polymerase and Functional Interactions of LigD with the DNA End-Binding Ku Protein. J. Biol. Chem. 285, 4815–4825. 10.1074/jbc.M109.073874 20018881 PMC2836087

[B60] Zhu H. Shuman S. (2005b). Novel 3′-Ribonuclease and 3′-Phosphatase Activities of the Bacterial Non-homologous End-Joining Protein, DNA Ligase D. J. Biol. Chem. 280, 25973–25981. 10.1074/jbc.M504002200 15897197

[B61] Zhu H. Shuman S. (2006). Substrate Specificity and Structure-Function Analysis of the 3′-Phosphoesterase Component of the Bacterial NHEJ Protein, DNA Ligase D. J. Biol. Chem. 281, 13873–13881. 10.1074/jbc.M600055200 16540477

[B62] Zhu H. Wang L. K. Shuman S. (2005). Essential Constituents of the 3′-Phosphoesterase Domain of Bacterial DNA Ligase D, a Nonhomologous End-Joining Enzyme. J. Biol. Chem. 280, 33707–33715. 10.1074/jbc.M506838200 16046407

