# Supplementary material for: LigD: A Structural Guide to the Multi-Tool of Bacterial Non-Homologous End Joining
Source: Front Mol Biosci. 2021 Nov 25;8:787709. doi: 10.3389/fmolb.2021.787709 (PMC8656161; doi:10.3389/fmolb.2021.787709)
Supplement: Supplementary file 1 [file Table1.DOCX]

Supplementary Material

**Supplementary Table 1. Experimental LigD domain structures and associated PDB accession numbers**

| LigD Domain | Species | PDB | Features |
| --- | --- | --- | --- |
| Ligase | *M. tuberculosis* | 6NHZ | Pre-adenylylation state |
| Ligase | *M. tuberculosis* | 1VS0 | Ligase-AMP intermediate |
| Ligase | *H. sapiens* | 6BKG | Catalytic core |
| Polymerase | *P. aeruginosa* | 2FAO | Apo |
| Polymerase | *M. tuberculosis* | 2IRU | Apo |
| Polymerase | *P. aeruginosa* | 2FAR | dATP-, manganese-bound |
| Polymerase | *P. aeruginosa* | 2FAQ | ATP-, manganese-bound |
| Polymerase | *M. tuberculosis* | 2IRY | dGTP-, manganese-bound |
| Polymerase | *M. tuberculosis* | 2IRX | GTP-, manganese-bound |
| Polymerase | *M. tuberculosis* | 3PKY | Pre-catalytic with DNA; UTP-, manganese-bound |
| Polymerase | *M. tuberculosis* | 4MKY | dsDNA break with complementary ends |
| Polymerase | *M. tuberculosis* | 2R9L | dsDNA break with non-complementary ends |
| Phosphoesterase | *P. aeruginosa* | 3N9B | Manganese-, sulfate anion-bound |
| Phosphoesterase | *P. aeruginosa* | 2LJ6 | Solution NMR structure |
| Phosphoesterase | *M. paludicola* | 5DMP | Archaeal homolog |
